# Body Shape Preferences: Associations with Rater Body Shape and Sociosexuality

**DOI:** 10.1371/journal.pone.0052532

**Published:** 2013-01-02

**Authors:** Michael E. Price, Nicholas Pound, James Dunn, Sian Hopkins, Jinsheng Kang

**Affiliations:** 1 Department of Psychology, School of Social Sciences, Brunel University, Uxbridge, United Kingdom; 2 School of Engineering and Design, Brunel University, Uxbridge, United Kingdom; McMaster University, Canada

## Abstract

There is accumulating evidence of condition-dependent mate choice in many species, that is, individual preferences varying in strength according to the condition of the chooser. In humans, for example, people with more attractive faces/bodies, and who are higher in sociosexuality, exhibit stronger preferences for attractive traits in opposite-sex faces/bodies. However, previous studies have tended to use only relatively simple, isolated measures of rater attractiveness. Here we use 3D body scanning technology to examine associations between strength of rater preferences for attractive traits in opposite-sex bodies, and raters’ body shape, self-perceived attractiveness, and sociosexuality. For 118 raters and 80 stimuli models, we used a 3D scanner to extract body measurements associated with attractiveness (male waist-chest ratio [WCR], female waist-hip ratio [WHR], and volume-height index [VHI] in both sexes) and also measured rater self-perceived attractiveness and sociosexuality. As expected, WHR and VHI were important predictors of female body attractiveness, while WCR and VHI were important predictors of male body attractiveness. Results indicated that male rater sociosexuality scores were positively associated with strength of preference for attractive (low) VHI and attractive (low) WHR in female bodies. Moreover, male rater self-perceived attractiveness was positively associated with strength of preference for low VHI in female bodies. The only evidence of condition-dependent preferences in females was a positive association between attractive VHI in female raters and preferences for attractive (low) WCR in male bodies. No other significant associations were observed in either sex between aspects of rater body shape and strength of preferences for attractive opposite-sex body traits. These results suggest that among male raters, rater self-perceived attractiveness and sociosexuality are important predictors of preference strength for attractive opposite-sex body shapes, and that rater body traits –with the exception of VHI in female raters– may not be good predictors of these preferences in either sex.

## Introduction

Mate preferences have been widely studied in many species, and much empirical work has focused on the importance of particular phenotypic traits as determinants of mating success. The extent to which mate preferences might vary systematically among individuals according to aspects of their own phenotype has received comparatively much less attention. However, there is now accumulating evidence that in many species, individuals (most commonly females) exhibit condition-dependent mate preferences, with the highest quality females exhibiting the strongest preferences for indicators of quality in potential mates [1], [2]. The predominant explanations have been based on the idea that choice itself is costly [3] and consequently choosiness can be a condition-dependent life-history trait [4].

In humans, research on condition-dependent preferences has focused on the role of traits such as an individual’s own (1) attractiveness and (2) sociosexuality (i.e. interest in short-term sexual relationships) in determining what they find attractive in others and how strong these preferences are (i.e. the degree of choosiness). Regarding the first type of condition-dependence above, why would the nature and strength of individuals’ preferences vary according to their own attractiveness? An evolutionary perspective and empirical evidence suggest that certain traits are attractive because they provide visual, auditory or olfactory cues to health and/or genetic quality (in both sexes) and fertility in females [5]. Given the signalling value of these traits, preferences for them (i.e. views on what is attractive) should generally be shared by members of a population. As a consequence of these shared preferences, assortative mating can arise even in the absence of any specific preference for partner similarity [6], [7] and positive assortment for attractiveness does seem to be a feature of human relationships [8]. Consequently, although one would be better off, all else equal, with as attractive a mate as possible, if one’s ability to acquire and retain an attractive mate will be limited by one’s own attractiveness, then pursuit of highly attractive partners by less attractive individuals could involve considerable wasted mating effort and costly losses in mating competition [9]. Less attractive individuals could avoid such costs by simply placing less weight on physical attractiveness when judging potential partners, while not adjusting their perception of how physically attractive others are [10]. Or alternatively, these costs could be avoided through facultative adjustments to the kinds of physical traits that one finds attractive, and to the strengths of one’s preferences for particular traits.

Sexually dimorphic traits have been found to be important determinants of attractiveness for bodies [11] and faces [12]–[14]. Men generally tend to prefer femininity in female faces [12] and bodies [15]–[19]. However, patterns of female preferences for masculine features in males are more complex. For example, female preferences for masculinity in male faces appear to vary systematically across the menstrual cycle, according to temporal context, female partnership status [20]–[22] and even according to the national level of socioeconomic inequality [23]. In light of findings such as these, studies of individual differences in mate preferences have often assessed attractiveness in terms of sex-typicality and have also tended to focus on variations in the preferences of females. For example, women who are rated (by self or others) as more attractive express increased attraction towards masculine faces [24] and voices [25]; and female raters who have lower (i.e., more feminine and attractive) waist-hip ratios also express an increased preference for masculine [26], [27], as well as healthy-looking [28], male faces. Evidence also suggests that masculine traits are preferred more in the context of short-term as opposed to long-term mating. In short-term mating contexts, women may tend to prioritize “good genes” and testosterone-related characteristics such as social dominance and masculine facial and body shape, whereas in long-term mating contexts they put more emphasis on perceived prosociality and willingness to invest in offspring [22], [29]. Consistent with this view, women report greater attraction to masculine bodies [30], [31], faces [22], [31], voices [31], [32], and scents [31] in the context of short-term as opposed to long-term mating.

In comparison with the number of studies focusing on the relationship between the attractiveness of a female and her mate preferences, relatively few studies have examined this relationship in males. Some evidence does suggest, however, that similarly to women, men who are more attractive and sex-typical in some respects exhibit stronger preferences for attractive and sex-typical female traits: Welling et al. [33] found that strength of attraction to femininity in female faces increased in men when their testosterone levels were high, and Burriss et al. [34] found that more attractive men expressed greater attraction to feminine faces, but only in short-term mating contexts. This latter result underlines another male-female similarity: both sexes exhibit stronger preferences for sex-typical (often physical) traits in the context of short-term as opposed to long-term mating; also consistent with this view is the finding that men report greater attraction to feminine voices in short-term contexts [35]. This increased emphasis on physical appearance in general, and sexually dimorphic traits in particular, in short-term contexts may be a strategy to increase the likelihood of mating with a fertile female: feminine voice and facial appearance may indicate higher estrogen levels and thus fertility [36]. Men tend to weigh fertility cues relatively heavily in short-term contexts, while placing a greater emphasis on traits such as parenting skills and kindness in long-term contexts [37], [38]. The focus of fertility cues in short-term contexts presumably occurs because valuation of such cues advantaged men reproductively in ancestral environments; this does not imply, however, that when men pursue short-term relationships in modern environments they are consciously striving to reproduce. This focus on cues to fertility may also be why men tend to pay relatively more attention to bodily appearance as opposed to facial appearance–the former being the more important source of fertility cues–in short-term mating contexts [39], [40].

The greater emphasis that both men and women place on attractive, sex-typical traits in short-term mating contexts is related to the other kind of condition-dependence mentioned above: the relationship between sociosexuality and attractiveness preferences. If attractive, sex-typical traits are preferred more in short-term mating contexts, then they should also be preferred more by people who are more oriented towards short-term relationships, that is, people higher in sociosexuality [41]. Some evidence supports this view. Higher-sociosexuality women prefer more masculine male faces [29], [42] and more muscular male bodies [42]. Higher-sociosexuality men exhibit stronger preferences for attractive female body-mass index and waist-hip ratio [43]. Moreover, they allocate more attention to attractive opposite-sex others, but no such effect is found among women [44]. Finally, both men and women who are higher in sociosexuality show stronger preferences for more symmetrical female (but not male) faces [45].

In the study reported here, we investigated whether body attractiveness preferences are associated with a rater’s own attractiveness and sociosexuality. In contrast to most previous studies that have focused on associations between female rater characteristics (e.g. attractiveness) and their preferences for various opposite-sex traits, we investigate these associations in both female and male raters. Our novel methodology involved using a 3D body scanner both to create rich, realistic stimuli that excluded non-shape cues (e.g. skin colour/texture), and also to collect precise anthropometric data from both raters and stimuli models. For 118 raters and 80 stimuli models, we used this scanner to extract bodily measurements that have been associated with attractiveness and sex-typicality in previous studies (male waist-chest ratio, female waist-hip ratio, and volume-height index in both sexes). We also measured self-perceived attractiveness and sociosexuality among raters. We then analyzed whether raters who had more attractive body traits, higher self-perceived attractiveness, and higher sociosexuality scores exhibited stronger preferences for body characteristics in opposite sex targets that are generally considered to be attractive.

## Materials and Methods

Participants were 118 adults, including 56 males aged 18–41 years (*M = *22.66, *SD* = 4.61) and 62 females aged 18–38 years (*M = *21.31, *SD* = 4.40). Most were undergraduates at an English University who participated in exchange for participation pool credit and/or a copy of their 3D body scan. Ethical approval for the study had previously been obtained from the Ethics Committee of the Brunel University School of Social Sciences, and all participants signed an informed consent form indicating their willingness to participate. After completing a questionnaire to measure sociosexuality and self-perceived attractiveness, participants changed into standardized, scanner-appropriate clothing (briefs and for females, a sports bra), and were body-scanned with an NX12 scanner, manufactured by TC^2^ (Cary, North Carolina, USA). This scanner uses white-light to create a 3D point cloud model of the body, and can generate hundreds of anthropometric measurements. According to the manufacturer, the scanner’s point accuracy is <1 mm, and its circumferential accuracy is <3 mm [46]. Participants stood erect in a standardized pose during the scan, without flexing any muscles, and with arms straightened and held slightly away from the sides the body. Two scans were obtained from each participant. For each trait, the two measurements were first used to assess repeatabilities, and were then averaged to produce the single measurement used to create predictors.

**Figure 1:**
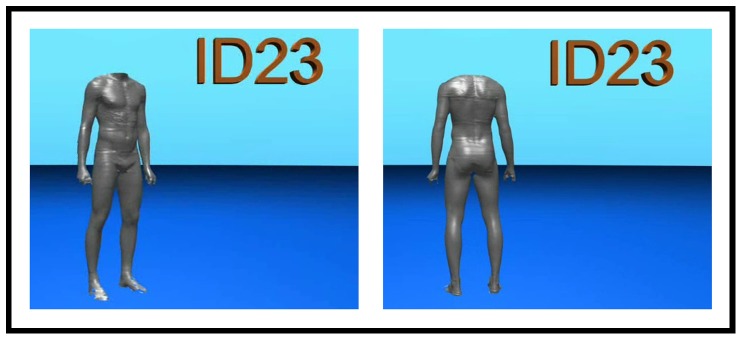
Example of body model stimuli (two frames of 360-degree rotation). Raters were presented with a video in which a rendered body model was rotated through 360 degrees over approximately 8 seconds. Figure depicts two frames from one of these videos.

After being body-scanned, participants rated the attractiveness of 40 opposite-sex body models (40 males, mean age 20.90±3.03 years; 40 females, mean age 20.50±2.36 years). These target body model stimuli (Figure 1) were created from body scans of people who had participated in a previous study involving the NX12 scanner, and like the participants in the current study, they wore only briefs and (if female) a sports bra for the scan. To create the stimuli, heads were removed from the body scan images and bodies were coloured gray. The same stimuli have been used in a previously published study [11].

### Variables

#### Anthropometric traits

Three anthropometric trait measurements were derived automatically from the body scan data, using NX12 software, for both the 118 participants (raters) and the 80 (40 of each sex) stimuli body models (targets). These were waist-hip ratio (WHR), waist-chest ratio (WCR) and volume-height index (VHI).

Waist-hip ratio (WHR) was defined as the narrowest waist circumference divided by widest hip circumference. WHR is a sexually dimorphic trait that is lower in females, and lower WHR is associated with increased female attractiveness in many populations, including UK university students [15]–[19]. Lower WHR may be regarded as more attractive because it is associated with increased fertility [47], [48] and health [15] in women, as well as with increased cognitive ability in both women and their offspring [49].

Waist-chest ratio (WCR) was defined as the narrowest waist circumference divided by widest chest circumference. Several studies indicate that lower-WCR (more V-shaped) male torsos are perceived as more attractive [11], [50]–[54]. A more V-shaped male torso may be regarded as more attractive because it is associated positively with testosterone, muscular development and physical dominance [55], [56].

Volume-height index (VHI) was based on measurements of body volume (excluding the head, in litres) and chin height (height from chin to the bottom of the feet, in metres) extracted by the body scanner’s NX12 software. To calculate VHI, we divided body volume by chin height squared, the same method used in previous research [57]. VHI has been shown to be an excellent predictor of attractiveness for the bodies of both females [57] and males [50]. VHI is closely related to the more widely used body mass index (BMI) which has been shown to predict attractiveness among both females [17], [57], [58] and males [52], [53]. However, research measuring both [50], [57] found VHI better predicted attractiveness than BMI, perhaps because it is by definition more closely related to the visible shape of a body. Body mass is highly correlated with body volume but the association is complicated by the differences in density between adipose and muscle tissue. For a given BMI, lean individuals will have a lower VHI than individuals with higher levels of adiposity. Associations between attractiveness and both VHI [50], [57] and BMI [17], [19], [59] are non-linear with attractiveness declining as VHI/BMI deviates either above or below an optimum. Accordingly, we have treated VHI as a non-linear predictor in our analyses.

Repeatabilities (intraclass correlation coefficients) for the five bodily measurements on which these traits were based (waist, hips, shoulders, volume, and chin height) ranged from.955 to.998. Missing scanned data made it impossible to measure WHR and VHI for four of the female body models, so in analyzing the attractiveness of these traits in the female body models, 36 rather than 40 models were used.

#### Body model attractiveness

Videos of the opposite-sex body models (e.g. Figure 1) were viewed by raters, with bodies presented one at a time in random order, with each body rotated 360 degrees so that the rater could comprehensively assess body shape attractiveness. Presentation time for each body model (i.e., duration of one complete rotation) was approximately eight seconds. Raters viewed each set of body models two separate times, and each time, they rated each body on a 100-mm scale from “unattractive” to “attractive”. In one viewing, raters were asked to rate each body based on how attractive it would be as a partner in a short-term relationship (STR), and in the other viewing, as a partner in a long-term relationship (LTR). The order in which the LTR and STR rating tasks occurred was counterbalanced across raters; there were no order effects (see below).

Before beginning each rating task, raters read definitions of “short-term relationship” (e.g., “a single date accepted on the spur of the moment, an affair within a long-term relationship, and possibility of a one-night stand”) and “long-term relationship” (“someone you may want to move in with, someone you may consider leaving a current partner to be with, and someone you may, at some point, wish to marry [or enter into a relationship on similar grounds as marriage]). These descriptions were identical to those used in a previous study [60]. Two of the 56 male participants were unavailable to provide body model ratings, so 54 males rated the female body models, whereas all 62 female participants rated the male body models.

#### Preference for attractive and sex-typical traits

For traits for which there was an overall linear association between the parameter and target attractiveness (WHR and WCR) we carried out separate linear regression analyses (SPSS 18.0) for each rater, with target scores on the trait of interest as the independent variable, and attractiveness ratings given by the rater as the dependent variable. The strength of each rater’s preference for a trait was defined as the slope of the attractiveness-trait regression function for that rater’s responses. For both WHR and WCR there was expected to be a general preference for low ratios, consequently increased preference strength would be indicated by more negative slope values while decreased preference strength would be indicated by less negative slope values (with values around zero indicating indifference). For the trait for which there was overall a non-linear (negative quadratic) association between the parameter and target attractiveness (VHI) we carried out separate curve estimation analyses (SPSS 18.0) for each rater. The extent to which each rater preferred low or high VHI targets was quantified by the vertex of the quadratic regression function for that rater where this yielded a biologically plausible function. Specifically, a negative quadratic with a VHI preference peak (vertex) above zero (i.e. for f(VHI) = a × VHI^2^+ b × VHI+c, a <0 and –b/2a ≥0).

#### Sociosexuality

We used the revised Sociosexual Orientation Inventory (SOI-R) [61], a well-validated measure of interest in short-term, uncommitted sexual relationships. The SOI-R consists of nine items, including three questions about number of past sexual partners (responses are on a nine-point scale from “zero” to “20 or more”), three statements measuring attitudes towards uncommitted sex (responses are on a nine-point scale from “strongly disagree” to “strongly agree”), and three questions about how frequently respondents experience desire for uncommitted sex (responses are on a nine-point scale from “never” to “at least once a day”). Scores on these nine items were summed to calculate SOI-R (α = .871; 95% CI [.833–.903]); the minimum possible score was 9, and the maximum possible score was 81.

#### Self-perceived attractiveness

To measure how physically attractive participants perceived themselves to be, we gathered responses on a nine-point scale from “very unattractive” to “very attractive”, to the item: “please tick the box indicating how physically attractive you think you are, in general”.

## Results

### Average Attractiveness of Target Body Traits to Raters

In accordance with theoretical considerations, and following from previous research, we have focused on WCR and VHI as predictors of male, and WHR and VHI as predictors of female, attractiveness. Prior to examining individual differences in rater preferences for these body traits, we first sought to establish the nature of the associations between these traits and how attractive each target body was perceived to be, on average, by raters. Reliability analyses revealed there was a high level of agreement among the 54 male raters when rating female body model attractiveness for both short term (α = .960; 95% CI [.937−.977]) and long term relationships (α = .966; 95% CI [.948−.980]), and also high agreement among the 62 female raters of male bodies (α = .982; 95% CI [.972−.989] and α = .977; 95% CI [.965−.987] for the two contexts respectively). Consequently, the average attractiveness of each target body was calculated for STR and for LTR. There were no order effects on relative target attractiveness arising from half the participants doing STR ratings first, and half doing LTR ratings first; correlations (Pearson’s r) between mean attractiveness ratings assigned to each target by the STR-first and LTR-first groups were all very high:.964; 95% CI [.933−.980] (males rating females for STR),.952; 95% CI [.911−.974] (males rating females for LTR),.971; 95% CI [.946−.984] (females rating males for STR), and.946; 95% CI [.900−.971] (females rating males for LTR).

Previous research has indicated that relationships between attractiveness and measures of body shape such as VHI [57], [62] and BMI [17], [19], [59] are not linear. Consequently, we first sought to establish whether higher order polynomial (quadratic or cubic) functions were a better fit than linear functions for the associations between target VHI and mean attractiveness ratings (mean of both STR and LTR attractiveness ratings given by all raters). For male bodies, the best fitting quadratic function (r^2^ = .409; F = 12.82; p<0.0001) modelled the association more closely than the best fitting linear (r^2^ = .195; F = 9.23; p<0.01) and cubic (r^2^ = .391; F = 11.87; p<0.001) functions. For female bodies, the best fitting quadratic function (r^2^ = .593; F = 24.10; p<0.0001) was also superior to the best fitting linear (r^2^ = .412; F = 23.86; p<0.0001) and cubic (r^2^ = .588; F = 23.52; p<0.0001) functions. Parameter estimates for these functions are presented in the Supporting Information (Tables S1 and S2) along with models computed separately for STR and LTR attractiveness.

**Figure 2:**
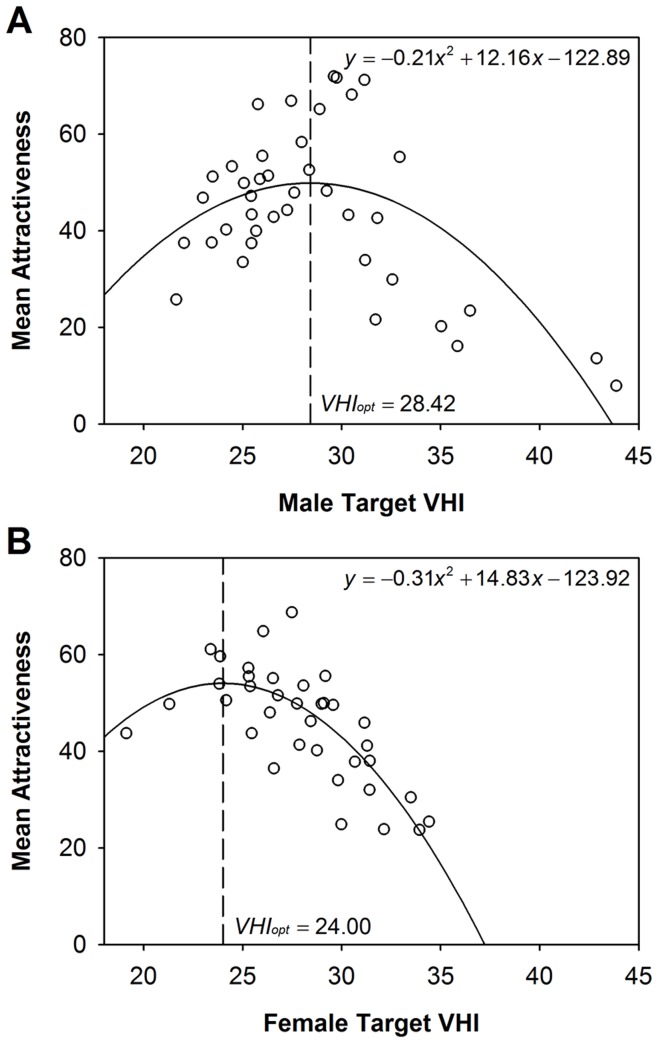
Association between target VHI and mean target attractiveness. Associations between target VHI and mean attractiveness ratings (mean of both STR and LTR attractiveness ratings given by all raters). (a) For male bodies, the best fitting quadratic function (r^2^ = .409; F = 12.82; p<0.0001) modelled the association more closely than the best fitting linear and cubic functions. (b) For female bodies, the best fitting quadratic function (r^2^ = .593; F = 24.10; p<0.0001) was also superior to the best fitting linear and cubic functions. These negative quadratic functions indicated that, as a predictor of mean attractiveness, the optimal VHI (VHI_opt_) is 28.42 for males and 24.00 for females (the vertices of the quadratic functions).

**Figure 3:**
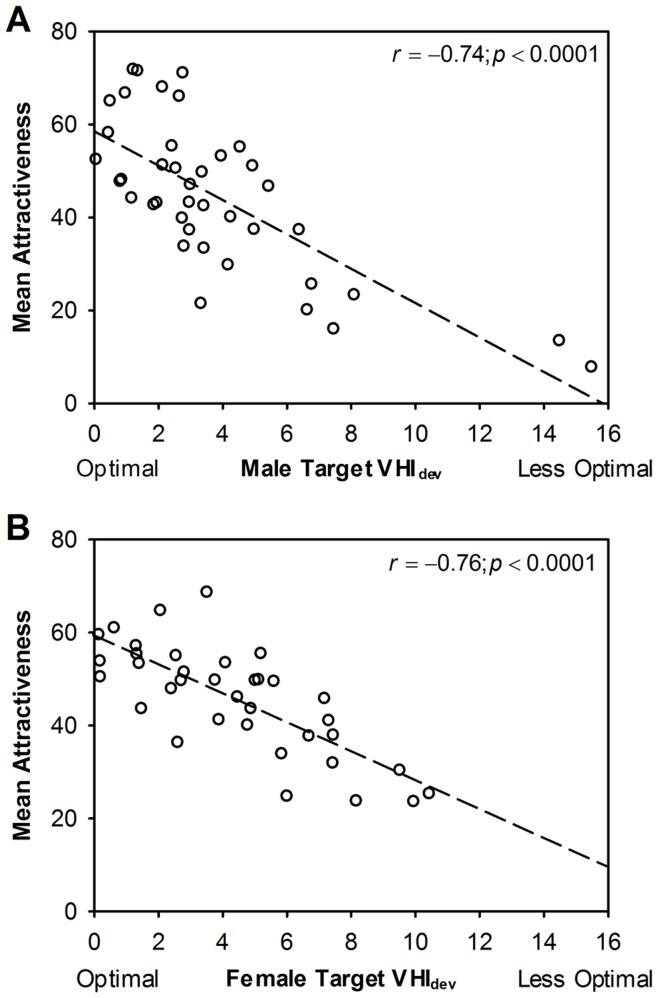
Association between target absolute deviation from sex-specific VHI optimum and mean target attractiveness. For each target, its absolute deviation from the sex-specific VHI optimum was computed (VHI_dev = _|VHI−VHI_opt_|). These deviation from optimality scores are linearly related to mean attractiveness (average of the STR & LTR attractiveness ratings given by all opposite sex raters) for (a) male targets (r = −735; n = 40; p<0.0001) and for (b) female targets (r = −762; n = 36; p<0.0001) with higher scores being associated with lower attractiveness. The deviation scores have been used in subsequent analyses so that a dimension based on VHI can be considered as a linear predictor along with other variables.

These negative quadratic functions (Figure 2) indicated that, as a predictor of mean attractiveness, the optimal VHI (VHI_opt_) is 28.42 for males and 24.00 for females (the vertices of functions). Given these values it is possible to compute, for each target body, its absolute deviation from the sex-specific VHI optimum (VHI_dev = _|VHI−VHI_opt_|), and this deviation from optimality score is linearly related (Figure 3) to mean attractiveness for males (r = −735; p<0.0001) and for females (r = −762; p<0.0001) with higher scores being associated with lower attractiveness. The deviation scores have been used in subsequent analyses so that a dimension based on VHI can be considered as a linear predictor alongside other variables.

For male targets, multiple regression analyses were used to examine the extent to which WCR and VHI deviation from optimality (VHI_dev_) each predicted attractiveness (either STR or LTR) when considered simultaneously in a model. The overall model explained 71.8% of the variance for STR attractiveness (R^2^ = .718, F(2,37) = 47.11, p<.0001) and 73.6% for LTR attractiveness (R^2^ = .736, F(2,37) = 51.53, p<. 0001). The contributions of the individual predictors to each model are shown in Table 1. As predicted, male attractiveness was negatively associated with WCR (i.e. positively associated with increasing chest, relative to waist, circumference) and with VHI_dev_. For female targets, a similar approach was taken to examine the extent to which WHR and VHI_dev_ each predicted attractiveness in each context (STR and LTR). The overall model explained 66.6% of the variance (R^2^ = .666, F(2,33) = 32.91, p<.0001) for STR attractiveness, and 62.9% for LTR attractiveness (R^2^ = .629, F(2,33) = 27.94, p<.0001). As predicted, attractiveness was negatively associated with WHR, but deviation from an optimal VHI was a better predictor of attractiveness for both relationship types. The contributions of the individual predictors to each model are shown in Table 1.

**Table 1:** Standardized coefficients (β) for body traits as predictors of attractiveness in linear regression analyses for each sex and relationship type.

Sex of Target Body	RelationshipType	BodyTrait	β	*t*	*p*
Male	STR	WCR	−.532	−4.98	<0.0001
		VHI_dev_	−.420	−3.93	<0.001
	LTR	WCR	−.539	−5.21	<0.0001
		VHI_dev_	−.425	−4.10	<0.001
Female	STR	WHR	−.275	−2.46	<0.05
		VHI_dev_	−.658	−5.89	<0.0001
	LTR	WHR	−.318	−2.70	<0.05
		VHI_dev_	−.601	−5.10	<0.0001

Notes: STR = Short-term relationship; LTR = Long-term relationship; WHR = Waist-hip ratio; WCR = Waist-chest ratio; VHI = Volume-height index; VHI_dev_ = absolute deviation from sex-specific optimal VHI (VHI_dev = _|VHI−VHI_opt_|). Values of *p* are two-tailed.

There were intercorrelations between the various body trait variables, with a bivariate association between WCR and VHI_dev_ in males (r = .578; n = 40; p<0.0001) and between WHR and VHI_dev_ in females (r = .435; n = 36; p<.01). However, multicollinearity was not a significant problem in the regression models with the Variance Inflation Factor (VIF) being 1.50 and 1.23 for the male and female models respectively. Moreover, Kolmogorov-Smirnov tests indicated that none of the predictor variables (WCR and VHI_dev_ in males, and WHR and VHI_dev_ in females) deviated significantly from normality.

### Individual Rater Characteristics and Variation in Preferences for Target Body Traits

Descriptive statistics for rater characteristics (body traits, self-perceived attractiveness, sociosexuality scores, and preference functions) are presented in the Supporting Information (Table S3). Also included in Supporting Information are analyses of (1) the extent to which raters’ self-perceived attractiveness scores accurately reflected their attractiveness as assessed anthropometrically (men were found to be more accurate than women; see Text S1), and (2) relationships between height and condition dependent preferences in males (none were found; see Text S2).

The preliminary analysis above indicated that the predictors of average target attractiveness to raters were extremely similar in both STR and LTR, and these ratings were highly consistent with average measure ICC (3,1) computed for consistency = 0.987; 95% CI [.979−.991]. In addition, average measure ICC (3,1) values were computed for each rater to assess the consistency of the STR and LTR attractiveness ratings given to the targets. Overall, when classified [63] consistency was good (0.60–0.74) or excellent (>0.75) for 71.1% of raters, and at least fair (0.40–0.59) for 86.4% of raters (median ICC values were 0.733 for male and 0.726 for female raters). Consequently, subsequent analyses of rater specific preferences have focused on mean attractiveness ratings (i.e. the average of the STR and LTR attractiveness ratings given by each rater to each model). When considering target VHI as a predictor of attractiveness ratings, curve estimation yielded quadratic functions with a VHI preference peak (vertex) above 0 for 51/54 male and 51/62 female raters.

To examine associations between individual male rater characteristics and variation in preferences for target female body, two separate multiple linear regression analysis were conducted with rater VHI deviation from optimality (VHI_dev_), rater WCR, rater self-perceived attractiveness and rater SOI-R considered simultaneously as predictors. The dependent variable in the first analysis was rater VHI preference peak (i.e. quadratic function vertex), and in the second analysis strength of WHR preference (i.e. WHR-Attractiveness function slope). Cases with missing values required for a regression were excluded from these analyses only, giving N = 51 and N = 49 for the two analyses respectively.

For male raters (Table 2), men with higher SOI-R scores exhibited stronger preferences for traits generally considered to be attractive in female targets (low VHI and low WHR). Specifically, SOI-R scores were significant predictors of both male rater preferences for low VHI (*β* = −.31, *t*(44) =  −2.31, *p*<.05) and also preferences for low WHR (*β = *−.33, *t*(46) =  −2.42, *p*<.05) in female targets. Moreover, self-perceived attractiveness was also a significant predictor of VHI preference peak with high self-perceived attractiveness being associated with a preference for low VHI in female targets (*β* = −.35, *t*(44) =  −2.54, *p*<.05).

**Table 2:** Linear regression with male rater characteristics considered as predictors of rater preferences for female target body traits (VHI and WHR).

	VHI Preference Peak (Quadratic Function Vertex), *n* = 49			WHR Preference (Slope of WHR-Attractiveness Function), *n* = 51		
Rater Characteristic	*Beta*	t	Sig	*Beta*	t	Sig
VHI_dev_	.076	.537	n.s.	.129	.906	n.s.
WCR	−.159	−1.157	n.s.	−.151	−1.094	n.s.
SP Attractiveness	−.354	−2.535	p<0.05	−.222	−1.559	n.s.
SOI-R	−.306	−2.307	p<0.05	−.326	−2.420	p<0.05

Notes: Analyses examine predictors of mean attractiveness preferences (mean of target long-term and short-term relationship attractiveness). WHR = Waist-hip ratio; WCR = Waist-chest ratio; VHI = Volume-height index; VHI_dev_ = absolute deviation from sex-specific optimal VHI (VHI_dev = _|VHI−VHI_opt_|); SP Attractiveness = self-perceived attractiveness; SOI-R = Sociosexuality Orientation Inventory (revised). Values of *p* are two-tailed. Overall model statistics: VHI Preference Peak - R^2^ = .259, F(4,44) = 3.85, p<0.01; WHR Preference Strength -; R^2^ = .208, F(4,46) = 3.02, p<0.05. All tolerances >.85 and variance inflation factors (VIF) <1.2 so multicollinearity is not an issue.

Neither rater VHI deviation from optimality (VHI_dev_) nor rater WCR were significant predictors of rater preferences for target VHI or target WHR. (In our initial analysis, male raters with higher VHI_dev_ did appear to display a significantly reduced strength of preference for attractive (low) WHR, as predicted. However, this relationship was due to a single influential outlier [Cook’s distance = .979 for the bivariate association between these variables]; consequently this case is excluded from the WHR preference regression reported in Table 2).

**Figure 4:**
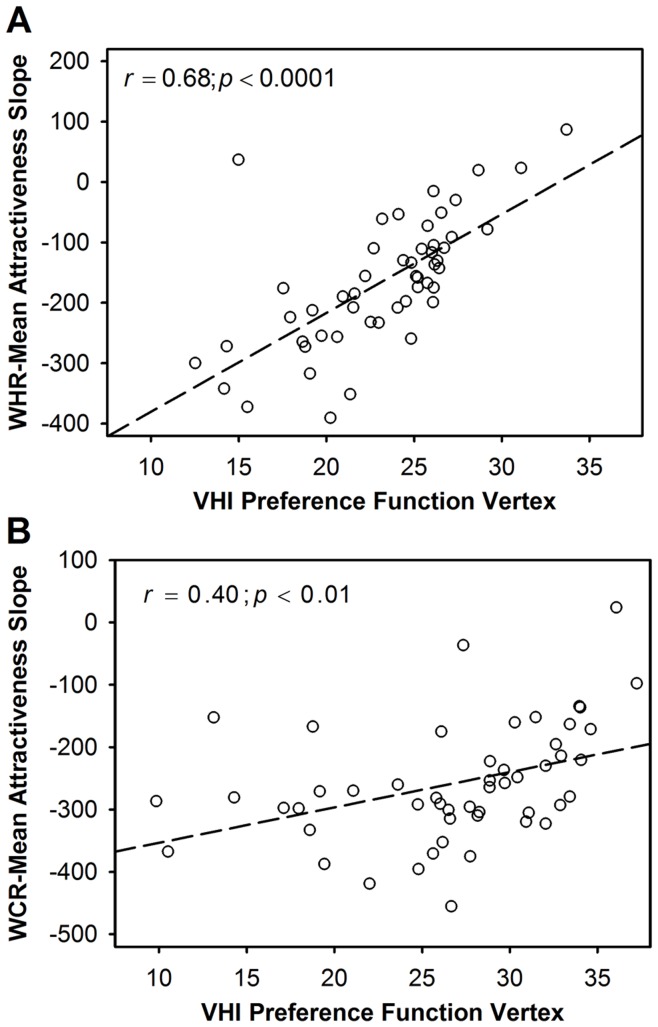
Association between rater specific VHI preference peaks and individual preference slopes for; (a) WHR for male raters and (b) WCR for female raters. The association between individual raters’ VHI quadratic preference function vertices and (a) for male raters their individual WHR-Mean attractiveness preference function slopes (unstandardized regression coefficients), and (b) for female raters their WCR-Mean attractiveness preference function slopes (unstandardized regression coefficients). For both, as slopes increase towards zero this indicates weaker preferences for low WHR or WCR. Consequently, associations shown here indicate that preference for high VHI is associated with reduced strength of preference for low WHR in male raters, and for low WCR in female raters. All functions are based on mean of STR and LTR attractiveness ratings given by each rater.

The apparent preference of some men for female targets with higher VHIs could be interpreted as a positive preference for larger women, or simply as less choosiness on the part of the raters, i.e. a weaker preference for a consensually desired trait in females (low VHI). Consistent with the second explanation is the finding that (considering mean of STR and LTR attractiveness judgements) male raters who exhibit a high VHI preference peak show a weaker preference (r = .68; n = 51; p<0.0001) for low WHR in female targets (Figure 4A). This arises not because they show a positive preference for high WHR, but because men with high VHI preference peak are more indifferent to WHR in female targets. No individual male raters exhibited significant WHR-attractiveness judgement associations with r >0, and the mean WHR-attractiveness slope for the male raters in the upper quartile of the VHI preference peak distribution (−58.30±95% CI[−109.03, −7.58]) was still significantly below 0 (one sample t = 2.56; df = 10; p<0.05).

To examine associations between individual female rater characteristics and variation in preferences for target male body, two separate multiple linear regression analysis were conducted with rater VHI deviation from optimality (VHI_dev_), rater WHR, rater self-perceived attractiveness and rater SOI-R considered simultaneously as predictors. The dependent variable in the first analysis was rater VHI preference peak (i.e. quadratic function vertex), and in the second analysis strength of WCR preference (i.e. WCR-Attractiveness function slope). Cases with missing values required for a regression were excluded from these analyses only giving N = 51 and N = 62 for the two analyses respectively.

For female raters (Table 3), there was only one significant association, with women with VHIs that are more attractive to men exhibiting stronger preferences for attractive male WCR. Specifically, female rater VHI deviation from optimality (VHI_dev_) was a significant predictor of weaker preferences for low WCR in male targets (*β* = .32, *t*(57) = 2.24, *p*<.05). None of the other rater characteristics entered were significant predictors of WCR preferences and none of the rater characteristics were significant predictors of VHI preference peaks.

**Table 3:** Linear regression with female rater characteristics considered as predictors of rater preferences for male target body traits (VHI and WCR).

	VHI Preference Peak (Quadratic Function Vertex), *n* = 51			WCR Preference (Slope of WCR-Attractiveness Function), *n* = 62		
Rater Characteristic	*Beta*	t	Sig	*Beta*	t	Sig
VHI_dev_	.130	.752	n.s.	.316	2.235	p<0.05
WHR	.069	.404	n.s.	−.092	−.654	n.s.
SP Attractiveness	.048	.327	n.s.	−.087	−.689	n.s.
SOI-R	.065	.438	n.s.	.012	.093	n.s.

Notes: Analyses examine predictors of mean attractiveness preferences (mean of target long-term and short-term relationship attractiveness). WHR = Waist-hip ratio; WCR = Waist-chest ratio; VHI = Volume-height index; VHI_dev_ = absolute deviation from sex-specific optimal VHI (VHI_dev = _|VHI−VHI_opt_|); SP Attractiveness = self-perceived attractiveness; SOI-R = Sociosexuality Orientation Inventory (revised). Values of *p* are two-tailed. Overall model statistics: VHI Preference Peak, R^2^ = .036, F(4,46) = 0.428, n.s.; WCR Preference Strength, R^2^ = .088, F(4,57) = 1.381, n.s. All tolerances >.70 and variance inflation factors (VIF) <1.4 so multicollinearity is not an issue.

It is noteworthy that female raters varied more in their VHI preferences than did male raters. Specifically, there was greater variance in VHI preference peaks for females (Levene’s test; F(1,100) = 5.24, p<0.05). Although none of the rater characteristics considered were significant predictors of VHI preference peaks for female raters, some of this variation could plausibly reflect facultative adjustments in general rater choosiness, or variation in preferences for specific body types. For example, the apparent preference of some women for male targets with higher VHIs could be interpreted as a positive preference for larger men, or simply as reduced rater choosiness, i.e., a weaker preference for a consensually desired lower VHI. Consistent with the second explanation is the finding that female raters who exhibit a high VHI preference peak show a weaker (but still directional) preference (r = .40; n = 51; p<0.01) for low WCR in male targets (Figure 4B). No individual female raters exhibited significant WCR-attractiveness judgement associations with r >0, and the mean WCR-attractiveness slope for the female raters in the upper quartile of the VHI preference peak distribution (−176.03±95% CI[−230.46, −121.61]) was still significantly less than 0 (one sample t = 7.12; df = 11; p<0.0001). These findings suggest that lack of choosiness generalises across traits.

## Discussion

The findings reported here provide some evidence of condition dependent variation in preferences among males. Specifically, the regression analysis examining predictors of male rater preferences indicated that males with higher sociosexuality scores showed stronger preferences for attractive (low) female VHI and WHR, and that males with higher self-perceived attractiveness showed stronger preferences for attractive (low) female VHI. However, neither VHI nor WCR in male raters were significantly related to strength of preference for low VHI or WHR in female bodies. There was less evidence of condition dependent variation in preferences among females. Females with more attractive VHI showed stronger preferences for attractive (low) WCR in male targets. However, no other female rater characteristics (WHR, self-perceived attractiveness, or sociosexuality) were significant predictors of strength of preference for attractive male VHI or WCR.

Although these results were in many ways consistent with expectations, they were also somewhat surprising in that there was more evidence of condition-dependent body shape preferences among males than among females. Previous research on condition-dependent mate preferences in both humans and non-human species has tended to focus on choices made by females [1], [2], [24]–[27]. This focus on female preferences has been influenced by parental investment and sexual selection theory [64], [65], which predicts that the sex higher in obligate parental investment–in most species, the female–will be choosier about mate selection. However, given the relatively high importance of male parental investment in humans, and the fact that physical attractiveness tends to be a more important aspect of female mate value than of male mate value [37], it is not surprising that men would exhibit significant choosiness about women’s physical attractiveness. Moreover, given that in general females in many species tend to be more discriminating, it may be the case that there will be more condition-dependent variation in choosiness among males because only the highest quality males can afford to turn down mating opportunities.

As noted, female WHR and self-perceived attractiveness failed to predict female preference strength for attractive male VHI and WCR. These results were surprising, as they seem inconsistent to some extent with reported positive relationships between attractive female WHR and/or self-perceived attractiveness, and strength of female preference for attractive/healthy-looking/masculine male faces/voices [24]–[28]. However, none of these prior studies used strength of female preference for attractive/masculine male body shape as outcome variables, which makes our results less directly comparable to theirs.

With regard to the relationship between sociosexuality and the strength of attractiveness preferences, our results are consistent with previous research [43] suggesting that higher-sociosexuality men have stronger preferences for attractive female bodies. Our results are also complementary with studies suggesting that higher-sociosexuality men pay more attention to attractive women [44] and exhibit stronger preferences for more symmetrical female faces [45]. However, as our results did not evidence a relationship between female sociosexuality and strength of attractiveness preferences, they were out of step with prior research suggesting that higher-sociosexuality women have stronger preferences for more masculine male faces and bodies [29], [42]. Reasons for this low complimentariness with earlier research are unclear, but future studies will hopefully cast additional light on the relationship between sociosexuality and attractiveness preferences in females.

We found good levels of consistency between ratings of attractiveness for short-term and long-term relationships, and consequently only included mean attractiveness ratings in our analysis of individual rater preferences. However, with other methodologies (e.g. between-subjects comparisons of STR and LTR attractiveness ratings) and other stimuli (e.g. faces, voices, bodies without other non-shape cues removed) it might be possible to obtain greater discrimination between STR and LTR preferences. A further limitation of our study is that we did not ask raters to state their sexual orientation. Although there is no reason to expect that the large majority of our participants were not heterosexual, the inclusion of data from some non-heterosexual raters could have limited our ability to detect strong preference patterns among heterosexual raters.

In conclusion, results from our study provide some evidence of the importance of rater characteristics as predictors of preference strength for attractive and sex-typical traits in opposite sex body shapes. Specifically, in males high sociosexuality and self-perceived attractiveness were associated with greater strength of preference for attractive traits when judging female bodies, and in females having an attractive (low) VHI was associated with expressing stronger preferences for low WCR in male bodies. In these respects our findings are consistent with earlier research suggesting that these predictors relate to preference strength for a variety of opposite-sex phenotypic traits. Our results, together with those of other recent studies [33], [34] also suggest that condition-dependent preferences in males, although not previously as widely studied as in females, are likely to have a significant role in human mating and are consequently in need of further investigation.

## Supporting Information

Table S1 Associations between male target body (n = 40) volume-height index (VHI) and attractiveness ratings (mean of ratings given by 62 female raters) for short-term relationship (STR), long-term relationship (LTR) and mean attractiveness.
(DOCX)

Table S2 Associations between female target body (n = 40) volume-height index (VHI) and attractiveness ratings (mean of ratings given by 54 male raters) for short-term relationship (STR), long-term relationship (LTR) and mean attractiveness.
(DOCX)

Table S3 Descriptive statistics for rater characteristics (body traits, sociosexuality scores and preference functions).
(DOCX)

Text S1 **Male height as a predictor of condition-dependent preferences.**
(DOCX)

Text S2 **Relationships between self-perceived and anthropometric measures of attractiveness.**
(DOCX)
