# Supplementary material for: Body Shape Preferences: Associations with Rater Body Shape and Sociosexuality
Source: PLoS One. 2013 Jan 2;8(1):e52532. doi: 10.1371/journal.pone.0052532 (PMC3534680; doi:10.1371/journal.pone.0052532)
Supplement: Table S1 — Associations between male target body (n = 40) volume-height index (VHI) and attractiveness ratings (mean of ratings given by 62 female raters) for short-term relationship (STR), long-term relationship (LTR) and mean attractiveness. (DOCX) [file pone.0052532.s001.docx]

| Attractiveness Type | Equation | Model Summary | | | | |  | Parameter Estimates | | | |
| --- | --- | --- | --- | --- | --- | --- | --- | --- | --- | --- | --- |
|  |  | R Square | F | df1 | df2 | p |  | Constant | b1 | b2 | b3 |
| STR | Linear | .198 | 9.36 | 1 | 38 | < .01 |  | 86.371 | -1.469 |  |  |
|  | Quadratic | .396 | 12.13 | 2 | 37 | < .0001 |  | -116.582 | 11.746 | -.208 |  |
|  | Cubic | .378 | 11.25 | 2 | 37 | < .001 |  | -42.810 | 4.868 | .000 | -.002 |
| LTR | Linear | .189 | 8.88 | 1 | 38 | < .01 |  | 85.847 | -1.426 |  |  |
|  | Quadratic | .418 | 13.27 | 2 | 37 | < .0001 |  | -129.799 | 12.617 | -.221 |  |
|  | Cubic | .399 | 12.29 | 2 | 37 | < .0001 |  | -52.171 | 5.345 | .000 | -.002 |
| Mean | Linear | .195 | 9.23 | 1 | 38 | < .01 |  | 86.175 | -1.450 |  |  |
|  | Quadratic | .409 | 12.82 | 2 | 37 | < .0001 |  | -122.894 | 12.164 | -.214 |  |
|  | Cubic | .391 | 11.87 | 2 | 37 | < .001 |  | -47.254 | 5.096 | .000 | -.002 |

Note. 2-tailed p-values.
