# Supplementary material for: Body Shape Preferences: Associations with Rater Body Shape and Sociosexuality
Source: PLoS One. 2013 Jan 2;8(1):e52532. doi: 10.1371/journal.pone.0052532 (PMC3534680; doi:10.1371/journal.pone.0052532)
Supplement: Table S2 — Associations between female target body (n = 40) volume-height index (VHI) and attractiveness ratings (mean of ratings given by 54 male raters) for short-term relationship (STR), long-term relationship (LTR) and mean attractiveness. (DOCX) [file pone.0052532.s002.docx]

| Attractiveness Type | Equation | Model Summary | | | | |  | Parameter Estimates | | | |
| --- | --- | --- | --- | --- | --- | --- | --- | --- | --- | --- | --- |
|  |  | R Square | F | df1 | df2 | p |  | Constant | b1 | b2 | b3 |
| STR | Linear | .438 | 26.48 | 1 | 34 | < .0001 |  | 107.939 | -2.170 |  |  |
|  | Quadratic | .612 | 25.99 | 2 | 33 | < .0001 |  | -112.890 | 14.167 | -.297 |  |
|  | Cubic | .606 | 25.37 | 2 | 33 | < .0001 |  | -41.274 | 6.110 | .000 | -.004 |
| LTR | Linear | .381 | 20.90 | 1 | 34 | < .0001 |  | 103.179 | -2.120 |  |  |
|  | Quadratic | .568 | 21.69 | 2 | 33 | < .0001 |  | -136.936 | 15.644 | -.323 |  |
|  | Cubic | .563 | 21.22 | 2 | 33 | < .0001 |  | -59.415 | 6.903 | .000 | -.004 |
| Mean | Linear | .412 | 23.86 | 1 | 34 | < .0001 |  | 105.606 | -2.146 |  |  |
|  | Quadratic | .593 | 24.08 | 2 | 33 | < .0001 |  | -123.924 | 14.834 | -.309 |  |
|  | Cubic | .588 | 23.52 | 2 | 33 | < .0001 |  | -49.636 | 6.469 | .000 | -.004 |

Note. 2-tailed p-values.
