# Supplementary material for: Body Shape Preferences: Associations with Rater Body Shape and Sociosexuality
Source: PLoS One. 2013 Jan 2;8(1):e52532. doi: 10.1371/journal.pone.0052532 (PMC3534680; doi:10.1371/journal.pone.0052532)
Supplement: Table S3 — Descriptive statistics for rater characteristics (body traits, sociosexuality scores and preference functions). (DOCX) [file pone.0052532.s003.docx]

|  | Males | | |  | Females | | | |  | Sex Difference | |
| --- | --- | --- | --- | --- | --- | --- | --- | --- | --- | --- | --- |
|  | n | Mean | S.D. |  | n | Mean | S.D. | |  | t | p |
| VHI | 56 | 28.28 | 3.77 |  | 62 | 27.89 | | 4.53 |  | 0.50 | .618 |
| WHR | 56 | 0.80 | 0.05 |  | 62 | 0.71 | | 0.03 |  | 12.70 | <.001 |
| WCR | 56 | 0.78 | 0.04 |  | 62 | 0.78 | | 0.03 |  | -0.78 | .436 |
| Self-Perceived Attractiveness | 53 | 6.17 | 1.05 |  | 62 | 6.10 | | 1.20 |  | 0.35 | .731 |
| SOI-Behaviour | 55 | 8.62 | 5.48 |  | 62 | 7.06 | | 4.17 |  | 1.74 | .085 |
| SOI-Attitude | 55 | 17.29 | 6.83 |  | 62 | 11.40 | | 6.98 |  | 4.60 | <.001 |
| SOI-Desire | 55 | 14.22 | 5.69 |  | 62 | 8.63 | | 4.53 |  | 5.91 | <.001 |
| SOI-Total | 55 | 40.13 | 13.86 |  | 62 | 27.10 | | 12.86 |  | 5.28 | <.001 |
| VHI preference peak | 51 | 23.17 | 4.43 |  | 51 | 26.73 | | 6.59 |  | 3.21 | <.001 |
| WHR attractiveness slope | 54 | -390.54 | 116.01 |  | - | - | | - |  |  |  |
| WHR attractiveness intercept | 54 | -92.92 | 85.17 |  | - | - | | - |  |  |  |
| WCR attractiveness slope | - | - | - |  | 62 | -506.06 | | 103.25 |  |  |  |
| WCR attractiveness intercept | - | - | - |  | 62 | 4.25 | | 84.00 |  |  |  |

Note. 2-tailed p-values. VHI = volume-height index; WHR = waist-hip ration; WCR = waist-chest ratio; SOI = Sociosexual Orientation Inventory. VHI preference peak = mean (STR & LTR) attractiveness vs target VHI quadratic preference function vertex. WHR attractiveness slope and intercept = mean (STR & LTR) attractiveness vs target WHR regression function parameters. WCR attractiveness slope and intercept = mean (STR & LTR) attractiveness vs target WCR regression function parameters. Slopes = unstandardized betas.
