# Supplementary material for: Body Shape Preferences: Associations with Rater Body Shape and Sociosexuality
Source: PLoS One. 2013 Jan 2;8(1):e52532. doi: 10.1371/journal.pone.0052532 (PMC3534680; doi:10.1371/journal.pone.0052532)
Supplement: Text S1 — Male height as a predictor of condition-dependent preferences. (DOCX) [file pone.0052532.s004.docx]

**Male height as a predictor of condition-dependent preferences**

A reviewer suggested that we include male height as a predictor of condition-dependent preferences. We did measure male height (to top of the head, via stadiometer) when processing participants. However, given concerns about multiple testing, we are hesitant to use height as a predictor in the main analysis, especially because its expected effects are unclear. We are not confident that absolute male height should be expected to have a positive linear relationship with male attractiveness, although the evidence does suggest that women tend to prefer men who are taller than themselves (Shepperd & Strathman, 1989; Hensley, 1994). By some measures, tall men seem to have more mating success than short men (e.g. Nettle, 2002), but this success could be the result of either intrasexual competition or intersexual choice. It does seem likely that very tall men are considered more attractive than very short men, but not clear that they are considered more attractive than men of average height (Jackson and Ervin 1992); for example, Hensley (1994) proposes a ‘ceiling effect’ whereby greater height stops being more attractive past six feet in an American sample. Most studies of male bodily attractiveness (such as those we cite in the manuscript) cannot easily measure the effect of height, because height is difficult to represent effectively in stimuli (e.g., individual bodies are usually shown, so relative height cannot be assessed).

For these reasons, the nature of the expected relationship between absolute height and attractiveness in males does not seem well-understood. We did check for linear and curvilinear relationships between the height of male raters and their preferences for attractive WHR and VHI in females, and found no evidence for these relationships (all *p*’s ≥.46).

**References**

Hensley, W. E. (1994). Height as a basis for interpersonal attraction. *Adolescence* 29: 469-474.

Jackson, L. A., & Ervin, K. S. (1992). Height stereotypes of women and men--the liabilities of shortness for both sexes. *Journal of Social Psychology* 132: 433-445.

Nettle, D. (2002). Height and reproductive success in a cohort of British men. *Human Nature* 13: 473-491.

Shepperd J. A. & Strathman A. J. (1989). Attractiveness and height: The role of stature in dating preference, frequency of dating, and perceptions of attractiveness. *Personality and Social Psychology Bulletin* 15: 617-627.
