# Supplementary material for: Body Shape Preferences: Associations with Rater Body Shape and Sociosexuality
Source: PLoS One. 2013 Jan 2;8(1):e52532. doi: 10.1371/journal.pone.0052532 (PMC3534680; doi:10.1371/journal.pone.0052532)
Supplement: Text S2 — Relationships between self-perceived and anthropometric measures of attractiveness. (DOCX) [file pone.0052532.s005.docx]

**Relationships between self-perceived and anthropometric measures of attractiveness**

There was a sex difference in the extent to which self-perceived attractiveness scores were consistent with anthropometric measures of attractiveness. Correlations between self-perceived attractiveness and WCR (r = -.25) and VHI_dev_ (r = -.30) were significant in the expected direction in male raters (n = 53, 1-tailed *p*’s < 0.05), whereas correlations between self-perceived attractiveness and WHR (r = .03) and VHI_dev_ (r < -.01) were nowhere near significance in female raters (n = 62). These results are consistent with a previous study (Brewer, Archer & Manning, 2007), which found female self-perceived attractiveness ratings to be inconsistent with male ratings of the females’ attractiveness.

**Reference**

Brewer, G., Archer, J., & Manning, J. (2007). Physical attractiveness: the objective ornament and subjective self-ratings. *Journal of Evolutionary Psychology* 5: 29–38.
